# Breastfeeding and its dimensions: an integrative review of health students’ knowledge

**DOI:** 10.1590/0034-7167-2024-0374

**Published:** 2025-11-28

**Authors:** Patricia Lima Pereira Peres, Maria Helena do Nascimento Souza, Thaís Emanuele da Conceição, Rosane Harter Griep, Rafael Braga Esteves, Antonella Nespoli, Donatella Valente, Giovanni Galeoto

**Affiliations:** I Universidade do Estado do Rio de Janeiro. Rio de Janeiro, Rio de Janeiro, Brazil; II Universidade Federal do Rio de Janeiro. Rio de Janeiro, Rio de Janeiro, Brazil; III Fundação Oswaldo Cruz. Rio de Janeiro, Rio de Janeiro, Brazil; IV Universidade de São Paulo. Ribeirão Preto, São Paulo, Brazil; V Università degli Studi di Milano Bicocca. Milão, Lombardia, Italy; VI Sapienza Università di Roma. Lácio, Roma, Italy

**Keywords:** Breastfeeding, Knowledge, Teaching, Health Science Students, Review., Lactancia Materna, Conocimiento, Enseñanza, Estudiantes de Ciencias de la Salud, Revisión.

## Abstract

**Objective:**

to analyze Brazilian and international scientific production on undergraduate health students’ knowledge about breastfeeding and the intrinsic dimensions of this topic.

**Method:**

an integrative review, carried out in the Embase, MEDLINE/PubMed, CINAHL, LILACS, Web of Science, Scopus, Cochrane and SciELO databases.

**Results:**

seventeen articles were identified. The analysis showed that, in most studies, students’ knowledge was restricted to the biological and management dimensions of lactation, while the sociocultural, legislation, breastfeeding protection policy and healthy complementary feeding dimensions were little explored and presented average knowledge.

**Conclusion:**

the weaknesses in students’ knowledge pointed to the importance of expanding studies on training future healthcare professionals regarding the multidimensionality of breastfeeding, with a view to improving learning of strategies aimed at promoting, protecting, and supporting breastfeeding.

## RESUMEN

**Objetivo:**

analizar la producción científica nacional e internacional sobre el conocimiento de estudiantes de grado en salud acerca de la lactancia materna y las dimensiones intrínsecas de este tema.

**Método:**

revisión integrativa, realizada en las bases de datos Embase, MEDLINE/PubMed, CINAHL, LILACS, Web of Science, Scopus, Cochrane y SciELO.

**Resultados:**

se identificaron 17 artículos. El análisis mostró que, en la mayoría de los estudios, el conocimiento de los estudiantes se restringió a las dimensiones biológicas y de gestión de la lactancia, mientras que las dimensiones socioculturales, la legislación, la política de protección de la lactancia materna y la alimentación complementaria saludable fueron poco exploradas y presentaron un conocimiento promedio.

**Conclusión:**

las fragilidades en el conocimiento de los estudiantes apuntaron la importancia de ampliar los estudios sobre la formación de los futuros profesionales de la salud acerca de la multidimensionalidad de la lactancia materna, con el fin de mejorar el aprendizaje de estrategias dirigidas a la promoción, protección y apoyo a la lactancia materna.

## INTRODUCTION

Breast milk is the ideal food for infant growth and development. Its nutritional value and importance are well-documented in national and international literature. The benefits of breastfeeding (BF) have long been recognized, and it is recommended for up to two years or more, exclusively for the first six months^(1,2)^.

In this regard, BF is recognized worldwide as a fundamental human right, and this means that all children, without distinction, have the right to be breastfed. However, this is not yet a reality, as less than half of the child population under six months old is exclusively breastfed^(3)^.

The causes for non-adherence to BF or even for early weaning are multifactorial. In the hierarchical theoretical model of factors associated with BF^(4)^, the determinants of BF are classified as structural (social and market context), environmental (integrated healthcare systems, family, community, employment, and workplace), and individual (related to the mother, baby, and their relationship). According to the authors, harmony among these contexts determines BF success or failure. Understanding these ecological or contextual factors is fundamental to developing promotion, protection, and support actions because it is based on the idea that all elements are interdependent and act in synergy.

BF is a feminine practice and part of the human repertoire, like eating, drinking, walking, and talking. In other words, BF depends much more on self-knowledge and the relationship between mother and baby than on health technology. BF is not a professional practice, but a human one. Therefore, the role of professionals and healthcare services is to provide support and conditions that allow mothers and babies to fully engage in BF with minimal compromise due to the aforementioned contexts.

Professional training in BF should incorporate an understanding of these contexts as determining factors, as well as strategies for dealing with them. In this sense, curricula addressing BF in different undergraduate health courses should incorporate the multiple dimensions involving the knowledge and practices of trainees so they can acquire the minimum common skills and competencies necessary to manage lactation and understand the intertwined social, commercial, and biological determinants, as well as the legislation and national policies that promote and protect BF when implemented. Although healthy complementary feeding (HCF) is a separate topic, due to its breadth, it goes hand in hand with BF. In this sense, HCF constitutes a dimension that should be incorporated into academic training.

Although national and international literature demonstrates the importance of professional support for BF mothers^(5-8)^, there is still a gap in knowledge about the type of knowledge that undergraduate health students acquire throughout their training and whether they are being prepared to provide the necessary assistance to families experiencing the BF process.

## OBJECTIVE

To analyze Brazilian and international scientific production regarding the knowledge of undergraduates in the health field about BF and the intrinsic dimensions of this topic.

## METHOD

### Study design

This is an integrative review, with a search protocol built from six stages^(9)^: 1) search question and objective elaboration; 2) database determination for the search for studies and inclusion and exclusion criteria; 3) data definition and extraction from the sample of selected publications; 4) sample assessment of selected studies; 5) analysis and discussion of results; 6) review presentation or synthesis of evidence found.

To compose the search question, the PICo strategy was used, an acronym for Population, Interest, Context^(10,11)^ (P: students, I: BF dimensions, Co: undergraduate studies in the health area). The guiding question was: which dimensions, intrinsic to BF, are addressed in the studies described in the literature that assess health undergraduate students’ knowledge?

### Search strategy

To develop the search strategy, an initial search was conducted in the MEDLINE database via PubMed to verify the controlled descriptors and synonyms, including the keywords identified in studies with similar topics to the review question. Controlled descriptors were selected from the Health Sciences Descriptors, Medical Subject Headings, and Medical Subject Headings (CINAHL vocabulary) to identify as many publications as possible that addressed the objectives of this review. Keywords were included in the search strategies, such as “assessment AND student knowledge OR student OR health student OR nursing student AND breastfeeding OR breastfeeding”, being used in Spanish for LILACS.

The search was conducted from October to December 2023, through the *Universidade de São Paulo* (USP) Digital Library and Collections Agency portal, connected by the USP VPN. No initial time frame was applied to the search, aiming at a greater scope of available scientific production. The articles included in the final sample were published between 1997 and 2021.

Subsequently, Chart 1 presents the search strategies and the sample number identified in the databases, respectively, for this review.

**Chart 1:** Search strategies performed in databases. Rio de Janeiro, RJ, Brazil, 2023

Database	Search strategies (syntax)	Sample (n=n)
CINAHL	tx (assessment or evaluation or “self-concept”) and and tx (“health student” or “nursing student” or “health students” or “nursing students” or “knowledge students”) and tx (“breast feeding” or “education breast feeding” or breastfeed or breastfeeding)	476
Scopus	(title-abs-key (assessment or evaluation or “self-concept”) and title-abs-key (“health student” or “nursing student” or “health students” or “nursing students” or “knowledge students”) and title-abs-key (“breast feeding” or “education breast feeding” or breastfeed or breastfeeding))	13
Embase	(title-abs-key (assessment or evaluation or “self-concept”) and title-abs-key (“health student” or “nursing student” or “health students” or “nursing students” or “knowledge students”) and title-abs-key (“breast feeding” or “education breast feeding” or breastfeed or breastfeeding))	47
Web of Science	(assessment or evaluation or “self-concept”) and (*todos os campos*) (“health student” or “nursing student” or “health students” or “nursing students” or “knowledge students”) and (*todos os campos*) “breast feeding” or “education breast feeding” or breastfeed or breastfeeding (*todos os campos*)	73
LILACS	(assessment or evaluation or “self-concept”) and (“health student” or “nursing student” or “health students” or “nursing students” or “knowledge students”) and (“breast feeding” or “education breast feeding” or breastfeed or breastfeeding)	77
PubMed	(assessment or evaluation or “self-concept”) and (“health student” or “nursing student” or “health students” or “nursing students” or “knowledge students”) and (“breast feeding” or “education breast feeding” or breastfeed or breastfeeding)	259
SciELO	(assessment or evaluation or “self-concept”) and (“health student” or “nursing student” or “health students” or “nursing students” or “knowledge students”) and (“breast feeding” or “education breast feeding” or breastfeed or breastfeeding)	23
Cochrane	(assessment or evaluation or “self-concept”) in title abstract keyword and (“health student” or “nursing student” or “health students” or “nursing students” or “knowledge students”) in title abstract keyword and (“breast feeding” or “education breast feeding” or breastfeed or breastfeeding) in title abstract keyword	0

### Study selection process

The studies included in the sample for analysis were selected after a database screening and subsequently exported to the Rayyan QCRI research tool. Rayyan QCRI is a multiplatform tool available online and as a free, cloud-based mobile application. It is designed to optimize and accelerate the initial screening of titles, abstracts, and keywords using an automated, intuitive, and highly usable system^(12)^.

### Data collection

After selecting the final 17 articles, the authors used a standardized form to extract the relevant data needed to answer the guiding question and objectives of the review. This form included information on study identification (author/year, country), methodological characteristics (method, design, sample, location, data collection instrument), main objective(s) and relevant findings classified according to the six thematic dimensions of BF defined for this review (sociocultural, biological, clinical management of lactation, BF policy, legislation and protection of BF, and HCF). The extracted data were subsequently organized and presented in a summarized form in synoptic charts for descriptive analysis and subsequent discussion.

### Selection criteria

Studies with primary data, indexed in databases in Portuguese, English and Spanish, published in journals with a peer review editorial policy, with title, abstracts and keywords were included.

Studies classified as gray literature, theses, dissertations, monographs, publications from scientific events, literature reviews regardless of typology and theoretical or methodological framework, articles that did not answer the research question, i.e., not related to the research topic, and duplicates were excluded.

Two independent reviewers carried out the process of selecting the articles for this integrative review. After the selection was complete, however, the two reviewers disagreed on the inclusion and exclusion criteria for three articles. The two reviewers then held a meeting and reached a consensus on the final sample, which included the three articles. This decision was made after a discussion and rigorous application of the established inclusion and exclusion criteria.

The results were presented in a synthesis chart (ID: author/year/country; method; objective(s); main findings; level of evidence; and a flowchart of the review selection stages) created by the authors for this integrative review.

The studies were classified according to the designations attributed by the authors using the JBI levels of evidence, which organize studies into five hierarchical levels based on methodological design. These levels range from experimental studies (level 1) to expert opinions (level 5). The authors classified the studies based on the methodological design reported in each primary study, following the JBI guidelines presented by Peters (2015). However, the specific criteria for determining the levels of evidence were not detailed, particularly with regard to the relationship between types of studies and their evidentiary hierarchy, as noted in the legend of Chart 2 ^(13)^.

**Chart 2:** Synthesis chart of studies included to compose the final sample of this integrative review (n = 17). Rio de Janeiro, RJ, Brazil,2023

ID Author/year/country	Method Design/sample/place	Objective(s)	Topic	Dimension	Level of evidence
Moura *et al*.^(16)^ 1997, Brazil	**Design:** cross-sectional and comparative between two groups, with descriptive, exploratory and quantitative characteristics. **Sample:** 90 undergraduate students (nutrition): 45 from San Jose State University, California, United States of America and 45 from *Pontifica Universidade Católica*, Campinas, Brazil. **Instrument:** questionnaire with short-answer questions. Four questions assess knowledge, asking for three answers each.	Compare knowledge and attitudes about BF.	Advantages of infant formula, advantages of BF, most common causes of BF failure and best age to introduce new foods.	Biological Sociocultural HCF	4.b
Kang *et al*.^(17)^ 2005, Korea	**Design:** cross-sectional and comparative. **Sample:** 341 university students, including those in the health field. **ICD:** 56 items modified from Williams and Hammer (1995), 47 (yes/no/do not know) and nine items (multiple choice), and 20 questions assessed attitude.	Explore differences in knowledge and attitudes about BF among college students according to gender and degree; and determine correlations between knowledge, attitudes and experiences related to BF.	Anatomy and physiology of lactation, benefits of BF, contraindications and barriers to BF, initiation of BF, prevention and management of sore nipples, infant assessments, use of BF aids, management of special situations and expression, and storage of breast milk.	Biological Management Sociocultural	4.b
Spear^(18)^ 2005, United States of America	**Design:** cross-sectional. **Sample:** 80 obstetric nursing students. Southeastern United States. **ICD:** modified 20-item version of Smith (2004) by replacing one knowledge question, adding questions on attitude, and seven questions characterizing participants.	Assess the basic knowledge about BF and selected attitudes of junior and senior baccalaureate nursing students; determine the need for inclusion of more in-depth information about BF in the undergraduate curriculum of obstetric nursing.	Properties of breast milk, milk production, the benefits of BF for both mother and baby, parameters of effective BF, maternal support, drugs and BF, weaning, stages of breast milk, jaundice, BF in public, weaning, and social support network.	Biological Management Sociocultural	4.b
Anjum ^(19)^ 2007, Pakistan	**Design:** cross-sectional. **Sample:** 344 firstand final-year medical students. **ICD:** pre-tested self-administered questionnaire with 22 questions.	Assess medical students’ knowledge at a private medical school about BF practices.	Initiation, duration and supplementation of BF, practice of BF and BF practices in special situations.	Biological Management	4.b
Junior-Lemos^(20)^ 2007, Brazil	**Design:** cross-sectional, randomly selected convenience sample. **Sample:** *Universidade Federal da Bahia*, nursing students: first year (100); last year (100) and medical students: first year (160), last year (160). **ICD:** questionnaire with 25 questions with yes/no/do not know answers, distributed in eight chunks of questions.	Investigate medical and nursing students’ knowledge on various issues involving BF, emphasizing the advantages for infants and mothers.	Physiology, benefits, properties of breast milk, recommendations and techniques, weaning, production/ejection, microorganisms, medications.	Biological Management Political	4.b
Ahmed^(21)^ 2011, Egypt	**Design:** descriptive exploratory study. **Sample:** 92 nursing students who completed didactic and clinical courses in maternal and child nursing. **ICD:** knowledge questionnaire on BF adapted from Brodribbs *et al*.	Assess BF knowledge, attitudes, and perceived adequacy of BF education among undergraduate nursing students in Cairo, Egypt.	Benefits of BF, maternal work, BF in public, introduction of food, physiology of lactation, clinical management, including maternal conditions that can affect BF, common BF problems and how to manage them.	Biological Management Sociocultural HCF	4.b
Ahmed^(22)^ 2011, United States of America	**Design:** descriptive exploratory study. **Sample:** convenience sample of 150 nursing students. **ICD:** the final questionnaire consisted of 24 items (three areas) and six questions about student demographics, nursing education methods, and nursing experience.	Assess the knowledge of BF among senior nursing students and identify the types of BF knowledge that students have; investigate the relationship between the different types of BF knowledge.	Benefits of BF (six items), physiology of BF (six items), management of BF, including maternal conditions that can affect BF and common BF problems and how to manage them (12 items).	Biological Management	4.b
Badagnan^(23)^ 2012, Brazil	**Design:** quantitative, observational, cross-sectional and descriptive study. **Sample:** students regularly enrolled in the second semester of the 1^st^ year (n=78) and 4^th^ year (n=75) of the bachelor’s degree in nursing at a public university in the state of São Paulo. **ICD:** questionnaire containing 25 questions about BF, with the possible answers “yes”, “no” and “do not know”. The questions were distributed in seven chunks.	Investigate the knowledge of BF among 1^st^ and 4^th^ year undergraduate nursing students in a bachelor’s degree program.	Physiology, benefits of BF, weaning, microorganisms, medications, recommendations, management and protection of BF.	Biological Management Sociocultural Political	4.b
Vandewark^(24)^ 2014, United States of America	**Design:** descriptive, observational, cross-sectional study. **Sample:** a group in their first semester of clinical nursing education (second semester) and one group in their last semester of nursing education (seniors)-at a mid-sized college of nursing. **ICD:** the attitudinal portion of this research was based on the 17 items taken from the II-FAS (Ahmed & El Guindy, 2011; de la Mora, Russell, Dungy, Losch, & Dusdieker, 1999; Riley, 2007). Three additional questions used by Ahmed and El Guindy (2011) were also asked to participants. A Likert scale was used.	Explore the relationship between knowledge and attitudes about BF among undergraduate nursing students at the beginning and end of their clinical training.	Benefits, physiology and management.	Biological Management Sociocultural	4.b
Aggarwal^(25)^ 2016, India	**Design:** cross-sectional, descriptive. **Sample:** 128 female Ayurvedic medical students. **ICD:** pre-tested and self-administered questionnaire with 12 questions.	Assess knowledge about practices, techniques and advantages of BF among medical students.	Technique, practices and advantages of BF.	Biological Management	4.b
Linares^(26)^ 2018, United States of America	**Design:** case-control. **Sample:** all nursing students, including undergraduate, DNP, and PhD students at a local state university in Kentucky (N = 793). **ICD:** 50-item questionnaire, including six questions on general demographic characteristics and 44 questions on BF knowledge.	Determine habits and knowledge about infant feeding; identify misconceptions about BF among nursing students; compare BF attitudes and knowledge among nursing students who had received a class on human lactation versus those who had not.	Infant feeding practices, opinions and basic knowledge such as “BF is beneficial for the mother and reduces the risk of disease”, “women with small breasts do not produce enough milk” and “BF and formula provide the same benefits”.	Biological Management Sociocultural	3.d
Bem Natan^(27)^ 2018, Israel	**Design:** cross-sectional and descriptive. **Sample:** 200 Israeli students from a large university in central Israel - 100 students from the nursing faculty and 100 from other faculties. **ICD:** questionnaire developed by Kavanagh *et al*. (2012), translated into Hebrew with permission, with 53 items, such as sociodemographic^(11)^, previous experience^(6)^, teaching support^(8)^, student attitudes^(12)^, intention to breastfeed^(3)^, institutional support^(2)^, Likert scale, knowledge of BF^(11)^ and yes/no response.	Assess attitudes and knowledge about human lactation in a group of nursing students.	Risk of obesity, superiority of BM over artificial formula, cost of BF, protective effect of BF, protection against infections/allergies, protective effect of BF on breast and cervical cancer.	Biological Sociocultural	4.b
Mohamad^(28)^ 2019, Malasya	**Design:** cross-sectional. **Sample:** 162 final-year medical and dental students from *Universiti Sains Malaysia* (75%). **ICD:** structured, self-administered questionnaire (11 items developed and validated by Che Muzaini *et al*.^(13)^), used with permission from the authors, with four parts: 1) questions about participants’ demographic characteristics; 2) participants’ future intentions to breastfeed and exposure to exclusive BF; 3) knowledge about BF; 4) students’ attitudes toward exclusive BF.	Assess the knowledge, attitudes, exposure and future intentions towards exclusive BF among final year medical and dental students at *Universiti Sains Malaysia*, Kelantan, Malaysia.	BF recommendations, advantages of BF (mother/child), BF problems (nipple trauma, engorgement, jaundice), BF duration and initiation and frequency, BM expression (expressing, storage and supply).	Biological Management Political Sociocultural	4.b
Altwalbeh^(29)^ 2021, Jordan	**Design:** descriptive cross-sectional study, convenience sampling. Sample: 72 obstetrics students **ICD:** short version of the Australian Breastfeeding Knowledge and Attitude Questionnaire (ABKAQ-SF), developed by Brodribb. Adapted questionnaire (48 items), with demographic characteristics of the students, their perceived confidence in their skills and their satisfaction regarding the BF content in the academic curriculum^(8)^, in addition to BF attitudes^(18)^, Likert scale, participants’ knowledge about BF^(22)^ with the answer type “correct/incorrect” and “do not know”, and 11 of them are false items.	Assess midwifery students’ knowledge and attitudes towards BF.	Benefits and physiology of BF and BF management.	Biological Management	4.b
Biggs^(30)^ 2020, United Kingdom	**Design:** cross-sectional, online collection. **Sample:** 411 5^th^ and 6^th^ year medical students from 22 universities in the United Kingdom. **ICD:** two online surveys, one to assess BF education provided in the curriculum (benefits of BF, difference between breast milk and formula) and another to determine students’ knowledge and perceptions regarding their confidence in supporting BF mothers, the perceived role of doctors in supporting BF, career aspirations and interest in receiving further education on BF.	Fill this knowledge gap and explore students’ perceptions of their readiness to support nursing.	Immunological properties, protection against infectious diseases, ovarian cancer, maternal breast, type I and II diabetes in adulthood, medical reasons for indicating the use of formula and environmental impact.	Biological Sociocultural	4.b
Cervera-Gasch^(31)^ 2021, Spain	**Design:** observational, descriptive, cross-sectional and multicenter study. **Sample:** 684 nursing students from three universities. **ICD:** AprendLac questionnaire, validated for Spanish, with 21 multiple-choice questions: 1) sociodemographic and academic; 2) knowledge.	Study nursing students’ nursing knowledge level in three Spanish public universities; explore which variables are related to the acquisition of this knowledge.	Recommendations for BF according to the WHO, artificial feeding, care for mastitis, virtual feeding, colostrum, BF in the first hour, feeding frequency, breast hygiene, weight loss in newborns, breast analgesia, assessment of inefficient sucking, attachment and positioning of the baby at the breast, lactogenesis, kangaroo care, benefits of BF for the baby and mother, and BF of premature babies.	Biological Management Political HCF	4.b
Yakovlev^(32)^ 2021, Russia	**Design:** multicenter study conducted at eight universities in Russia. **Sample:** 1,088 5^th^ and 6^th^ year medical students, interns and pediatric residents. **ICD:** questionnaire developed by Yakovlev et al. with 43 questions, 32 of which were closed, nine open and two partially closed.	Analyze (and its dynamics over the last decade) medical students’, interns’ and residents’ knowledge about the support and promotion of ML in the Russian Federation.	Preparation for BF, support for nursing mothers, misconceptions, physiology of lactation, infant feeding, WHO recommendations, stimulation of lactation, advertising of formula.	Biological Management Political Sociocultural HCF	4.b

*Note: ID - identification; ICD - instrument with dimensions; ML - maternal lactation; BF - breastfeeding; WHO - World Health Organization; HCF - healthy complementary feeding.*

*Level of evidence: 2e - Prospectively controlled quasi-experimental studies; 3d - Case-control study; 4b - Descriptive or observational cross-sectional study (Peters, 2015).*

In this study, knowledge of BF will be considered based on six dimensions, which encompass the multiplicity of aspects involved in BF practice success, such as lactation sociocultural, biological, clinical management, BF policy (national and international), legislation and BF protection, and HCF.

The authors classified the findings of the primary studies into six thematic dimensions based on a careful analysis of the studies’ objectives, data collection instruments, and reported results. Each study was categorized into the dimension(s) whose topics were the central focus of the investigation and results presented. Categorization sought the greatest possible alignment between the article’s content and the dimensions’ definitions proposed for this review.

The decision to classify the aforementioned dimensions is justified by the fact that it is a starting point for structuring a teaching methodology that is as comprehensive as possible, considering BF as an existential phenomenon that is constituted by the synergy between nature and culture^(14)^.

After categorizing the studies, the data were synthesized for a descriptive analysis according to year of publication, language, location where the study was conducted, objective, study design, and level of evidence. Finally, the articles obtained were analyzed and synthesized in a descriptive manner to support the presentation of the results, discussion, and conclusions of this review study.

## RESULTS

The database search yielded 967 articles. Of these, 96 were excluded as duplicates. Then, 871 articles were assessed based on titles and abstracts using the established criteria. This process identified 25 articles to be read in full while considering the inclusion and exclusion criteria. Ultimately, 17 articles were selected.

**Figure 1:**
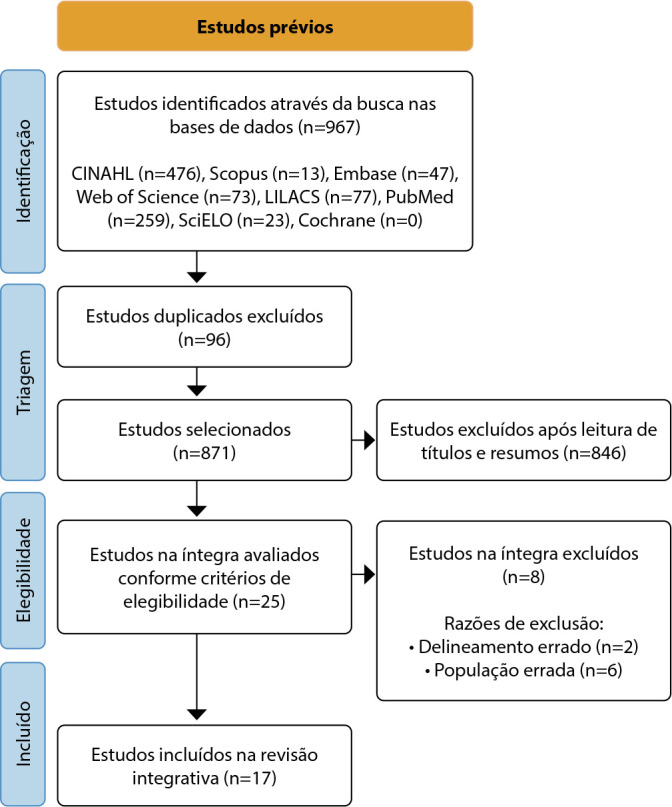
Flowchart of the stages for identifying, selecting and including studies, adapted from the Preferred Reporting Items for Systematic Reviews and Meta-Analyses

*Source: Page MJ et al.^(15)^*

The process of selecting the articles and the reasons for exclusion were presented using the flowchart created by the authors for this review (Figure 1):

Chart 2 presents the profile of the sample of articles selected for analysis.

As shown in Chart 2, the predominant countries among the included articles were the United States (four articles, 23%) and Brazil (three articles, 17%). In terms of continental representation, the Americas and Asia contributed seven articles each (41%), Europe contributed two (12%), and Africa contributed one (6%). English was the predominant language.

Regarding the type of data collection instrument, eight used their own questionnaires and nine reported having adapted the original instrument with authorization, namely AprendLac^(31)^, Brodribb^(21,24,29)^, Che Muzaine^(28)^, Kavanagh^(27)^, Smith^(18)^, and Wlliams and Hammer^(17)^.

As for the approach to dimensions, six articles addressed two dimensions^(21,23,28,31)^; six addressed three dimensions^(16-18,20,24,26)^; four addressed four dimensions^(20,22,27,30)^; and only one addressed five dimensions^(32)^. No questionnaire had a unidimensional approach, but the dimension with the highest occurrence of approach was the biological dimension, followed by lactation management.

Concerning the biological dimension, studies incorporate the importance of BF to prevent diseases in children^(21,23,24,27)^, aspects related to: anatomy and physiology of lactation^(17,20-24,29,31)^; benefits of breast milk^(17,18,20-24,28,29,31)^; maternal conditions that may affect BF^(7)^; use of drugs during lactation^(20,21,23,24)^; protective effect of BF in preventing breast and cervical cancer^(21,24,27)^; properties of breast milk^(18,20,31)^; superiority over artificial formula^(21,24,27)^; metabolic changes and risk of obesity^(27)^.

With a focus on such topics, researchers were concerned with identifying students’ knowledge about aspects related to breast function, the structures involved in lactation, the composition of human milk and lactation under special conditions so that future professionals can feel more confident and prepared for lactation management.

In this sense, studies that addressed the dimension of lactation management focused on topics such as: BF of premature infants^(31)^; BF and bonding^(24)^; assessment of inefficient sucking by infants^(17,31)^; barriers, difficulties and contraindications to BF^(16,17)^; weaning^(20,23)^; BF duration^(19,28)^; breast milk expression (milking, storage and supply)^(17,28)^; BF frequency^(16,21,28)^; breast hygiene^(31)^; BF initiation^(17,19,28)^; breast-feeding techniques^(17,19,20,22,25,29,31)^; mastitis management^(31)^; effective BF parameters^(18)^; latching and positioning of the baby at the breast^(25,31)^; newborn weight loss^(31)^; prevention and management of nipple trauma^(17,22,28)^; use of BF intermediaries/auxiliaries^(2)^; and BF supplementation^(16,19,21,24,31)^.

The approach to the political dimension was restricted to international policy, notably the World Health Organization (WHO) recommendation on BF duration, which is “exclusive BF for six months and continued for two years or more”^(20,23,28,31,32)^ and the Kangaroo Method^(31)^, which constitutes a policy aimed at qualifying the care of newborns and their families^(33)^.

The legislation dimension was not included in any of studies analyzed. Although, in some of them, the word “protection of BF” was present, it was associated with management or the sociocultural dimension. In the ten articles that included the sociocultural dimension in their instruments, the emphasis was on self-image “women with small breasts do not produce enough milk”^(26)^, BF as a human value^(23,26,28)^, advice on artificial feeding^(31)^, BF in public understood in light of the culture of a people^(16,18,21,24)^ and cost of BF^(27)^.

Concerning the HCF dimension, it was found that the studies were focused on the level of knowledge about food introduction^(16,21,24)^ and infant feeding management after the weaning period^(26)^.

## DISCUSSION

This review revealed that the majority of studies that met the inclusion criteria were conducted in Asia and the United States, and the evidence was obtained from cross-sectional quantitative research designs.

Although it is a government concern to establish curricular guidelines to guarantee students’ specific knowledge, skills and abilities throughout their academic training process^(34)^, it is noted that international and Brazilian scientific production regarding health undergraduates’ knowledge about BF in its multiple dimensions is still incipient.

Studies aimed at providing undergraduate students with knowledge about BF should begin with the understanding that adequate professional training and, consequently, safe and quality care require a minimum amount of relevant theoretical and practical content. This content should enable students to acquire the necessary skills and competencies to impact the success of BF upon graduation.

These findings underscore the urgent need for higher education institutions and health course boards to update their curricula to more deeply address the various dimensions of BF. The training of future professionals must go beyond biological and clinical management aspects to incorporate solid knowledge of the social, cultural, economic, legislative, and political determinants impacting the decision to breastfeed and BF practices. Integrating this knowledge with the Brazilian National Curricular Guidelines for health courses and the principles of the Brazilian Healthcare System (In Portuguese, *Sistema Único de Saúde* - SUS) will enable graduates to more effectively promote, protect, and support BF while considering the phenomenon’s complexity and families’ contexts. This requires adopting active, problematizing teaching methodologies that stimulate critical reflection and the development of interprofessional and collaborative skills.

From this perspective, it is evident that knowledge, skills, and attitudes must be grounded in the multifactorial BF paradigm, where different types of knowledge are interconnected. It is also important to consider that the ways in which we learn content in college are not linear or uniform.

This statement aligns with studies showing that teaching and learning methods are inherently related to the skills developed during training. These skills include cognitive skills, which involve acquiring knowledge from sources such as schools, books, universities, workplaces, media, and interpersonal interactions; practical skills, which involve knowing how to perform an action based on decision-making; and moral skills, which motivate individuals to apply knowledge based on their desires, beliefs, values, and experiences directed towards specific objects, situations, or people^(34,35)^

As a reflection of the above-described knowledge, issues related to lactation management are an important dimension because they cover the primary guidance and conduct that should be provided to BF mothers. Mothers may encounter problems or difficulties at the beginning of the lactation period or when trying to prevent early weaning. In addition to individual guidance, legislation and policies that support BF should be considered.

It was observed that studies examining the knowledge of health undergraduate students about BF with a focus on the political and legislative dimensions are still incipient. None of the analyzed studies considered the legislative dimension, and the political dimension was mostly restricted to WHO recommendations and the kangaroo method^(36)^. Therefore, it is crucial for future healthcare professionals to understand the strategies and actions included in national and international BF policies, as well as the legal foundations that aim to protect, promote, and support BF. Learning about the recommendations already established in decrees, laws and resolutions, such as the Brazilian Standard for the Marketing of Food for Infants and Young Children, maternity/paternity leave and the Brazilian Network of Human Milk Banks, are of paramount importance for the support and safe practice of BF, and are a framework for the development and implementation of effective policies and programs. Understanding the role of healthcare professionals in advocating for and enforcing this legislation is essential for creating an environment that is conducive to BF.

BF is like a prism that reveals new forms when projected upon. It can be understood from the perspectives of economics, religion, politics, the market, social relations, gender, science, ethics, and bioethics. These perspectives are intertwined in a web that converges towards BF’s success or disperses equidistantly. The investment we make as individuals, families, healthcare professionals, society, and the state will tip the scales in one direction or the other.

For this reason, it is essential to understand that the decision to breastfeed does not depend solely on knowledge of its benefits, or on physiological processes such as the functionality of the mother’s glands and the child’s stomatognathic system^(37)^.

From this perspective, it is worth considering the sociocultural dimension of BF, which refers to environmental factors, family traditions, social and daily practices, popular beliefs, and the influence of messages transmitted through media, literature, soap operas, films, and social media posts, all of which can impact a mother’s decision to breastfeed^(38)^. Studies that addressed this dimension highlighted aspects such as body image (“women with small breasts do not produce enough milk”), BF as a human value, and advice on artificial feeding and BF in public from a cultural perspective. These aspects underscore the importance of incorporating knowledge about sociocultural issues into the training of healthcare professionals. This approach fosters a broader understanding of BF, extending beyond the mother-baby dyad. Consequently, healthcare professionals can more effectively position themselves as members of the BF woman’s support network. Sensitivity to identify and address sociocultural barriers is crucial to providing individualized and respectful care.

Despite the focus on promoting exclusive BF in the first six months of a child’s life, students should be aware of the issues related to the HCF dimension^(16,21,39,40)^. The studies analyzed focused on knowledge of the correct time and form of food introduction, as well as the management of infant feeding after the weaning period. However, other crucial aspects of complementary feeding were little explored in the scientific production included in this review. These aspects include the nutritional quality of foods, family feeding practices, food and nutritional security, and the influence of sociocultural factors on the acceptance of certain foods.

These findings are corroborated by investigations with healthcare professionals, which point to the relevance of nutritional and hygiene guidelines. These guidelines must be provided to nursing mothers to ensure adequate nutrition after exclusive BF ends and, consequently, satisfactory growth and development during the first years of life^(39)^. Therefore, the training of future healthcare professionals must cover the different aspects of HCF more comprehensively to enable them to provide comprehensive, culturally sensitive guidance to families.

### Study limitations

One limitation of the present study is the small number of articles obtained from the databases searched. Additionally, the absence of a detailed protocol for assessing the methodological quality of the included studies and categorizing the findings into thematic dimensions is a limitation. However, the authors carefully classified the articles based on their content. Nevertheless, we believe these facts did not compromise the quality of the investigation. The results indicated the need for future studies to expand the discussion on undergraduate students’ knowledge, attitudes, and practices regarding BF.

### Contributions to nursing and health

Investing in the academic training of undergraduate nursing students and students from other health fields is important so they can practice active listening and provide support to families experiencing the BF process. This highlights the importance of course coordinators and professors prioritizing content relevant to the multidimensionality of BF practice. The importance of future health network professionals is also emphasized, as they will be responsible for providing support to mothers who wish to breastfeed.

## CONCLUSIONS

This integrative literature review analyzed scientific literature on health undergraduates’ knowledge of BF, based on the topic’s intrinsic dimensions. The studies addressed the guiding question and revealed that students’ knowledge was primarily limited to the biological and management aspects of lactation. In contrast, the sociocultural dimensions, HCF, legislation, and BF protection policy were rarely explored in the analyzed studies, revealing significant knowledge gaps among future professionals in these crucial areas.

It is considered that student training in BF should cover, in an equitable and in-depth manner, the six dimensions presented: biological; clinical management; legislation; BF protection policy; sociocultural; and HCF. Integrating this knowledge is fundamental to providing care that focuses not only on guidelines and techniques, but also on understanding the social, cultural, political, and legal context, as well as the multitude of factors involved in the BF process.

In light of the identified knowledge gaps, particularly in the least addressed dimensions, it is crucial for higher education institutions to promote curricular reviews that ensure the comprehensive and in-depth inclusion of BF in all its facets. Active and participatory teaching methodologies that encourage critical reflection, the development of practical skills, and sensitivity to the sociocultural and emotional issues surrounding BF should be incorporated. Integrating teaching about BF into current health policies is also essential to prepare students to act in accordance with national and international guidelines and become agents of change in promoting and protecting BF in the SUS.

The limited number of international and national publications assessing students’ knowledge of BF in all its dimensions underscores the need for future studies with diverse, robust methodological designs. These designs should include interventions and qualitative research to broaden understanding of the most effective teaching methods for undergraduate health courses and to investigate the impact of different pedagogical approaches on future professionals’ knowledge and skills.

## Data Availability

Research data is only available upon request.
