# Supplementary material for: Breastfeeding and its dimensions: an integrative review of health students’ knowledge
Source: Rev Bras Enferm. 2025 Nov 28;78(5):e20240374. doi: 10.1590/0034-7167-2024-0374 (PMC12673611; doi:10.1590/0034-7167-2024-0374)
Supplement: Translation

# Translation: Amamentação e suas dimensões: uma revisão integrativa sobre conhecimento de estudantes da área da saúde

**DOI:** 10.1590/0034-7167-2024-0374pt

**Authors:** Patricia Lima Pereira Peres, Maria Helena do Nascimento Souza, Thaís Emanuele da Conceição, Rosane Harter Griep, Rafael Braga Esteves, Antonella Nespoli, Donatella Valente, Giovanni Galeoto

**Affiliations:** I Universidade do Estado do Rio de Janeiro. Rio de Janeiro, Rio de Janeiro, Brasil; II Universidade Federal do Rio de Janeiro. Rio de Janeiro, Rio de Janeiro, Brasil; III Fundação Oswaldo Cruz. Rio de Janeiro, Rio de Janeiro, Brasil; IV Universidade de São Paulo. Ribeirão Preto, São Paulo, Brasil; V Università degli Studi di Milano Bicocca. Milão, Lombardia, Itália; VI Sapienza Università di Roma. Lácio, Roma, Itália

**Keywords:** Aleitamento Materno, Conhecimento, Ensino, Estudantes de Ciências da Saúde, Revisão.

## Abstract

**Objetivo:**

analisar a produção científica brasileira e internacional acerca do conhecimento de graduandos da área da saúde sobre aleitamento materno e as dimensões intrínsecas a esta temática.

**Método:**

revisão integrativa, realizada nas bases Embase, MEDLINE/PubMed, CINAHL, LILACS, *Web of Science*, Scopus, Cochrane e banco de dados SciELO.

**Resultados:**

foram identificados 17 artigos. A análise mostrou que, na maioria dos estudos, o conhecimento dos estudantes se restringiu às dimensões biológica e de manejo da lactação, enquanto as dimensões socioculturais, de legislação, de política de proteção ao aleitamento materno e de alimentação complementar saudável foram pouco exploradas e apresentaram um conhecimento regular.

**Conclusão:**

as fragilidades no conhecimento dos estudantes apontaram para a importância da ampliação de estudos sobre a formação dos futuros profissionais de saúde acerca da multidimensionalidade da amamentação, com vistas a melhorar a aprendizagem das estratégias voltadas à promoção, proteção e apoio do aleitamento materno.

## INTRODUÇÃO

O leite materno é o alimento ideal para o crescimento e o desenvolvimento infantil. Suas características nutricionais e importância são amplamente documentadas na literatura nacional e internacional. Não é de hoje que seus benefícios são reconhecidos e que a amamentação é recomendada até 2 anos ou mais, sendo exclusiva nos primeiros seis meses^(1,2)^.

Neste sentido, o aleitamento materno (AM) é mundialmente reconhecido como um direito humano fundamental, e isso significa que todas as crianças, indistintamente, têm direito a serem amamentadas. Contudo, isto ainda não é uma realidade, pois menos da metade da população infantil menor de seis meses está em AM exclusivo^(3)^.

As causas para a não adesão ao AM ou até mesmo para o desmame precoce são multifatoriais. No modelo teórico hierarquizado dos fatores associados ao AM^(4)^, os determinantes do AM estão classificados em estruturais (contexto social e de mercado), ambientais (sistemas integrados de saúde, a família, comunidade, emprego e local de trabalho) e individuais, que são aqueles relacionados à mãe e ao bebê e à relação entre eles. Para os autores, a harmonia entre esses contextos determina o sucesso ou não do AM. O entendimento desses fatores ecológicos ou contextuais é fundamental para o desenvolvimento de ações de promoção, proteção e apoio, uma vez que está baseado na ideia de uma engrenagem em que todos os elementos são interdependentes e agem em sinergia.

O AM é uma prática feminina, faz parte do repertório humano, assim como comer, beber, andar e falar. Ou seja, é algo que depende muito mais do conhecimento de si (mulher) e do outro (bebê) do que da tecnologia de saúde (medicalização). Não é uma prática profissional, mas humana; logo, o papel do profissional e dos serviços de saúde é de oferecer apoio e condições para que mães e bebês o façam plenamente, com o mínimo de comprometimento gerado por esses contextos citados anteriormente.

A formação profissional em AM deve incorporar a compreensão desses contextos como fatores determinantes, bem como estratégias para o seu enfrentamento. Nesse sentido, é importante que os currículos que tratam da temática AM nos diferentes cursos de graduação da área da saúde incorporem as múltiplas dimensões que envolvem os saberes e práticas do profissional em formação, pois assim ele poderá adquirir habilidades e competências mínimas e comuns capazes não apenas de manejar a lactação, mas também de compreender os determinantes sociais, comerciais e biológicos imbricados, a legislação e política nacional que, quando implementadas, promovem e protegem o AM. A alimentação complementar saudável (ACS), embora seja um tema à parte, devido à sua amplitude, caminha junto ao AM, e nesse sentido, constitui uma dimensão que deve ser incorporada na formação acadêmica.

Apesar de a literatura nacional e internacional demonstrar a importância do apoio profissional às mães que amamentam^(5-8)^, ainda há uma lacuna no conhecimento sobre o tipo de conhecimento que os estudantes de graduação da área da saúde adquirem ao longo de sua formação e se estes estão sendo preparados para prestar a assistência necessária às famílias que vivenciam o processo da amamentação.

## OBJETIVO

Analisar a produção científica brasileira e internacional acerca do conhecimento de graduandos da área da saúde sobre AM e as dimensões intrínsecas a esta temática.

## MÉTODO

### Tipo de estudo

Trata-se de revisão integrativa, com um protocolo de busca construído a partir de seis etapas^(9)^: 1) elaboração da pergunta de busca e objetivos; 2) determinação das bases de dados para a busca dos estudos e dos critérios de inclusão e exclusão; 3) definição e extração dos dados da amostra de publicações selecionadas; 4) avaliação da amostra de estudos selecionados; 5) análise e discussão dos resultados; 6) apresentação da revisão ou síntese das evidências encontradas.

Para composição da questão de busca, utilizou-se a estratégia PICo, acrônimo de População, Interesse, Contexto^(10,11)^ (P: estudantes, I: dimensões do tema AM, Co: graduação na área da saúde). A questão norteadora foi: quais dimensões, intrínsecas à temática da AM, são abordadas nos estudos descritos na literatura que avaliam o conhecimento de estudantes de graduação na área da saúde?

### Estratégia de busca

Para estruturar as estratégias de buscas, foi realizad a pesquisa inicial na base de dados *Medical Literature Analysis and Retrieval System Online* (MEDLINE), via *National Library of Medicine* (PubMed), para verificar os descritores controlados e sinônimos, incluindo palavras-chave identificadas em estudos com temáticas semelhantes à questão de revisão. Os descritores controlados foram selecionados nos Descritores em Ciências da Saúde, *Medical Subject Heading* e *MH Exact Subject Heading* (vocabulário CINAHL) para localizar o máximo de publicações que respondessem aos objetivos desta revisão. Foram incluídas palavras-chave nas estratégias de buscas, como “avaliação *AND* conhecimento de estudante *OR* estudante *OR* estudante da saúde *OR* estudante de enfermagem *AND* amamentação *OR* amamentação”, sendo utilizadas no idioma espanhol para a base LILACS.

A busca foi realizada de outubro a dezembro de 2023, por meio do portal da Agência de Bibliotecas e Coleções Digitais da Universidade de São Paulo (USP), conectado pelo VPN da USP. Não foi aplicado um recorte temporal inicial para a busca, visando uma maior abrangência da produção científica disponível. Os artigos incluídos na amostra final foram publicados no período de 1997 a 2021.

Na sequência, são apresentados no Quadro 1 as estratégias de buscas e o número da amostra identificado nas bases de dados, respectivamente, para a presente revisão.

**Quadro 1:** Estratégias de busca realizadas nas bases de dados. Rio de Janeiro, RJ, Brasil, 2023

Base de dados	Estratégias de busca (sintaxe)	Amostra (n=n)
CINAHL	*tx (assessment or evaluation or “self-concept”) and and tx (“health student” or “nursing student” or “health students” or “nursing students” or “knowledge students”) and tx (“breast feeding” or “education breast feeding” or breastfeed or breastfeeding)*	476
Scopus	*(title-abs-key (assessment or evaluation or “self-concept”) and title-abs-key (“health student” or “nursing student” or “health students” or “nursing students” or “knowledge students”) and title-abs-key (“breast feeding” or “education breast feeding” or breastfeed or breastfeeding))*	13
Embase	*(title-abs-key (assessment or evaluation or “self-concept”) and title-abs-key (“health student” or “nursing student” or “health students” or “nursing students” or “knowledge students”) and title-abs-key (“breast feeding” or “education breast feeding” or breastfeed or breastfeeding))*	47
*Web of Science*	*(assessment or evaluation or “self-concept”) and* (todos os campos) *(“health student” or “nursing student” or “health students” or “nursing students” or “knowledge students”) and* (todos os campos) *“breast feeding” or “education breast feeding” or breastfeed or breastfeeding* (todos os campos)	73
LILACS	*(assessment or evaluation or “self-concept”) and (“health student” or “nursing student” or “health students” or “nursing students” or “knowledge students”) and (“breast feeding” or “education breast feeding” or breastfeed or breastfeeding)*	77
PubMed	*(assessment or evaluation or “self-concept”) and (“health student” or “nursing student” or “health students” or “nursing students” or “knowledge students”) and (“breast feeding” or “education breast feeding” or breastfeed or breastfeeding)*	259
SciELO	*(assessment or evaluation or “self-concept”) and (“health student” or “nursing student” or “health students” or “nursing students” or “knowledge students”) and (“breast feeding” or “education breast feeding” or breastfeed or breastfeeding)*	23
Cochrane	*(assessment or evaluation or “self-concept”) in title abstract keyword and (“health student” or “nursing student” or “health students” or “nursing students” or “knowledge students”) in title abstract keyword and (“breast feeding” or “education breast feeding” or breastfeed or breastfeeding) in title abstract keyword*	0

### Processo de seleção dos estudos

A seleção dos estudos incluídos na amostra para análise foi realizada após a triagem nas bases de dados, sendo posteriormente exportados para a ferramenta de pesquisa Rayyan QCRI. Trata-se de multiplataforma, disponível tanto em formato *online* quanto como aplicativo móvel gratuito, baseada em nuvem. Essa ferramenta é projetada para otimizar e acelerar o processo de triagem inicial de títulos, resumos e palavras-chave, utilizando um sistema automatizado, intuitivo e de alta usabilidade^(12)^.

### Coleta de dados

Após a seleção final dos 17 artigos, os dados relevantes para responder à questão norteadora e aos objetivos da revisão foram extraídos por meio de um formulário padronizado elaborado pelos autores. Este formulário contemplou informações sobre a identificação do estudo (autor/ano, país), características metodológicas (método, desenho, amostra, local, instrumento de coleta de dados), objetivo(s) principal(ais) e achados relevantes classificados de acordo com as seis dimensões temáticas da amamentação definidas para esta revisão (sociocultural, biológica, manejo clínico da lactação, política de AM, legislação e proteção do AM, e ACS). Os dados extraídos foram subsequentemente organizados e apresentados de forma sintetizada em quadros sinópticos para análise descritiva e posterior discussão.

### Critérios de seleção

Foram incluídos estudos com dados primários, indexados nas bases de dados nos idiomas português, inglês e espanhol, publicados em periódicos com política editorial de revisão por pares, com título, resumos e palavras-chave.

Foram excluídos estudos classificados como literatura cinzenta, teses, dissertações, monografias, publicações oriundas de eventos científicos, revisões de literatura independente da tipologia e referencial teórico ou metodológico, artigos que não respondessem à questão de pesquisa, ou seja, não relacionados ao tema da pesquisa, e duplicados.

O processo de seleção dos artigos desta revisão integrativa foi realizado por dois revisores independentes, após a finalização da seleção três artigos divergiram, quanto à inclusão e exclusão feita pelos dois revisores. Neste sentido, os dois revisores realizaram uma reunião e decidiram pela amostra consenso, incluindo os três artigos, na amostra final, após discussão e aplicação rigorosa dos critérios de inclusão e exclusão estabelecidos.

Os resultados foram apresentados com quadro sintético dos achados (ID: autor/ano/ país; método; objetivo(s); achados principais (dimensões); nível de evidência; e fluxograma com as etapas da seleção da revisão), criado pelos autores para a presente revisão integrativa.

A classificação dos estudos seguiu as denominações atribuídas pelos autores, utilizando os níveis de evidência do JBI, que organiza os estudos em cinco níveis hierárquicos baseados no delineamento metodológico. Os níveis incluem desde estudos experimentais (nível 1) até opiniões de especialistas (nível 5). A classificação foi realizada pelos autores com base no delineamento metodológico reportado em cada estudo primário incluído, seguindo as diretrizes do JBI apresentadas por Peters (2015). No entanto, os critérios específicos para determinar os níveis de evidência não foram detalhados, especialmente quanto à relação entre os tipos de estudo e sua hierarquia evidencial, conforme mencionado na legenda do Quadro 2 ^(13)^.

**Quadro 2:** Quadro-síntese dos estudos incluídos para compor a amostra final da presente revisão integrativa (n = 17). Rio de Janeiro, RJ, Brasil, 2023

ID Autor/ano/país	Método Desenho/amostra/local	Objetivo(s)	Temática	Dimensão	Nível de evidência
Moura *et al*.^(16)^ 1997, Brasil	**Desenho:** transversal e comparativo entre dois grupos, com características descritivas, exploratórias e quantitativas. **Amostra:** 90 graduandos (nutrição): 45 da *San Jose State University*, Califórnia, Estados Unidos da América e 45 da Pontifica Universidade Católica, Campinas, Brasil. **Instrumento:** questionário com perguntas de respostas curtas. Quatro perguntas avaliam conhecimento, pedindo três respostas cada.	Comparar conhecimentos e atitudes sobre amamentação.	Vantagens da fórmula infantil, vantagens da amamentação, causas mais comuns da falha na amamentação e melhor idade para introdução de novos alimentos.	Biológica Sociocultural ACS	4.b
Kang *et al*.^(17)^ 2005, Coreia	**Desenho:** transversal e comparativo. **Amostra:** 341 estudantes universitários, incluindo da área da saúde. **ICD:** 56 itens modificados de Williams e Hammer (1995), sendo 47 (sim/não/não sei) e nove itens (múltipla escolha), e 20 questões avaliavam a atitude.	Explorar as diferenças nos conhecimentos e atitudes dos estudantes universitários sobre a amamentação de acordo com o gênero e a graduação; e determinar correlações entre conhecimentos, atitudes e experiências relacionadas ao AM.	Anatomia e fisiologia da lactação, benefícios da amamentação, contraindicações e barreiras à amamentação, início da amamentação, prevenção e manejo dos mamilos doloridos, avaliações do lactente, uso de auxiliares de amamentação, manejo de situações especiais e expressão, e armazenamento de leite materno.	Biológica Manejo Sociocultural	4.b
Spear^(18)^ 2005, Estados Unidos da América	**Desenho:** transversal. **Amostra:** 80 estudantes de enfermagem obstétrica. Sudeste dos Estados Unidos. **ICD:** versão modificada de 20 itens de Smith (2004) por substituição de uma questão de conhecimento, acréscimo de questões sobre atitude e de sete questões de caracterização dos participantes.	Avaliar o conhecimento básico sobre AM e atitudes selecionadas de estudantes de enfermagem do bacharelado júnior e sênior; determinar a necessidade de inclusão de informações mais aprofundadas sobre AM no currículo do curso de graduação em enfermagem obstétrica.	Propriedades do leite materno, produção láctea, os benefícios da amamentação tanto para a mãe quanto para o bebê, os parâmetros de amamentação eficaz, apoio materno, drogas e amamentação, desmame, fases do leite materno, icterícia, amamentação em público, desmame, e rede social de apoio.	Biológica Manejo Sociocultural	4.b
Anjum ^(19)^ 2007, Paquistão	**Desenho:** transversal. **Amostra:** 344 estudantes de medicina do primeiro e último ano. **ICD:** questionário autoadministrado pré-testado com 22 questões.	Avaliar o conhecimento de estudantes de medicina de uma faculdade de medicina privada sobre as práticas de amamentação.	Início, duração e suplementação do AM, prática do AM e práticas do AM em situações especiais.	Biológica Manejo	4.b
Junior-Lemos^(20)^ 2007, Brasil	**Desenho:** transversal, amostra de conveniência selecionada aleatoriamente. **Amostra:** Universidade Federal da Bahia, estudantes de enfermagem: primeiro ano (100); último ano (100) e de medicina: primeiro ano (160), último ano (160). **ICD:** questionário com 25 questões com respostas sim/não/não sei, distribuídas em oito blocos de perguntas.	Investigar o conhecimento dos alunos dos cursos de medicina e enfermagem sobre várias questões que envolvem o AM, enfatizando as vantagens para as crianças e para as mães.	Fisiologia, benefícios, propriedades do leite materno, recomendações e técnicas, desmame, produção/ejeção, microrganismos, medicamentos.	Biológica Manejo Política	4.b
Ahmed^(21)^ 2011, Egito	**Desenho:** estudo exploratório descritivo. **Amostra:** 92 estudantes de enfermagem que completaram cursos didáticos e clínicos de enfermagem materno-infantil. **ICD:** questionário de conhecimento sobre AM adaptado de Brodribbs *et al.*	Avaliar o conhecimento sobre amamentação, atitudes e adequação percebida da educação sobre amamentação entre estudantes de enfermagem de bacharelado no Cairo, Egito.	Benefícios do AM, trabalho materno, amamentação em público, introdução alimentar, fisiologia da lactação, manejo clínico, incluindo condições maternas que podem afetar o AM, problemas comuns de amamentação e como manejá-los.	Biológica Manejo Sociocultural ACS	4.b
Ahmed^(22)^ 2011, Estados Unidos da América	**Desenho:** estudo descritivo exploratório. **Amostra:** amostra de conveniência com 150 estudantes de enfermagem. **ICD:** o questionário final consistiu em 24 itens (três áreas) e seis perguntas sobre a demografia dos estudantes, métodos de educação para o AM e experiência de AM.	Avaliar o conhecimento sobre AM entre estudantes seniores de enfermagem e identificar os tipos de conhecimento sobre AM que os estudantes possuem; investigar a relação entre os diferentes tipos de conhecimento sobre AM.	Benefícios da amamentação (seis itens), fisiologia da amamentação (seis itens), manejo da amamentação, incluindo condições maternas que podem afetar a amamentação e problemas comuns de amamentação e como administrá-los (12 itens).	Biológica Manejo	4.b
Badagnan^(23)^ 2012, Brasil	**Desenho:** estudo de abordagem quantitativa, observacional, transversal e descritivo. **Amostra:** estudantes regularmente matriculados no segundo semestre do 1º ano (n=78) e do 4º ano (n=75) do curso de bacharelado em enfermagem de uma universidade pública do estado de São Paulo. **ICD:** questionário contendo 25 perguntas sobre amamentação, com possibilidades de resposta “sim”, “não” e “não sei”. As perguntas estavam distribuídas em sete blocos.	Investigar os conhecimentos dos estudantes do 1º e 4º ano de um curso de bacharelado em enfermagem sobre AM.	Fisiologia, benefícios da amamentação, desmame, micro-organismos, medicamentos, recomendações, manejo e proteção ao AM.	Biológica Manejo Sociocultural Política	4.b
Vandewark^(24)^ 2014, Estados Unidos da América	**Desenho:** estudo descritivo, observacional, transversal. **Amostra:** um grupo em seu primeiro semestre de educação em enfermagem clínica (segundo semestre) e um grupo em seu último semestre de educação em enfermagem (sêniores) - em uma faculdade de enfermagem de médio porte. **ICD:** a parte de atitude desta pesquisa foi baseada nos 17 itens retirados do II-FAS (Ahmed & El Guindy, 2011; de la Mora, Russell, Dungy, Losch, & Dusdieker, 1999; Riley, 2007). Três perguntas adicionais utilizadas por Ahmed e El Guindy (2011) também foram feitas aos participantes. Utilizou-se a escala de Likert.	Explorar a relação entre conhecimentos e atitudes sobre AM em estudantes de graduação em enfermagem no início e no final de sua formação clínica.	Benefícios, fisiologia e manejo.	Biológica Manejo Sociocultural	4.b
Aggarwal^(25)^ 2016, Índia	**Desenho:** transversal, descritivo. **Amostra:** 128 estudantes de medicina Ayurvédica do sexo feminino. **ICD:** questionário pré-testado e autoadministrado com 12 perguntas.	Avaliar o conhecimento sobre práticas, técnicas e vantagens do AM entre estudantes de medicina.	Técnica, práticas e vantagens da amamentação.	Biológica Manejo	4.b
Linares^(26)^ 2018, Estados Unidos da América	**Desenho:** caso controle. **Amostra:** todos os estudantes de enfermagem, incluindo estudantes de graduação, DNP e PhD em uma universidade estadual local no Kentucky (N = 793). **ICD:** questionário com 50 itens, incluindo seis perguntas sobre características demográficas gerais e 44 perguntas sobre conhecimento em AM.	Determinar os hábitos e conhecimentos sobre alimentação infantil; identificar conceitos errôneos sobre AM entre estudantes de enfermagem; comparar atitudes de AM e conhecimento entre os estudantes de enfermagem que tinham recebido uma aula sobre lactação humana *versus* aqueles que não tinham.	Práticas de alimentação infantil, opiniões e conhecimentos básicos como “amamentar é benéfico para a mãe e reduz o risco de doenças”, “mulheres com seios pequenos não produzem leite suficiente” e “amamentação e fórmula dão os mesmos benefícios”.	Biológica Manejo Sociocultural	3.d
Bem Natan^(27)^ 2018, Israel	**Desenho:** transversal e descritivo. **Amostra:** 200 estudantes israelenses de uma grande universidade do centro de Israel - 100 estudantes da faculdade de enfermagem e 100 de outras faculdades. **ICD:** questionário construído por Kavanagh *et al*. (2012), traduzido para o hebraico com autorização, com 53 itens, como sociodemográfico^(11)^, experiência prévia^(6)^, apoio docente^(8)^, atitudes dos alunos^(12)^, intenção de amamentar^(3)^, apoio institucional^(2)^, escala Likert, conhecimento de AM^(11)^ e resposta sim/não.	Avaliar as atitudes e o conhecimento sobre a lactação humana em um grupo de estudantes de enfermagem.	Risco de obesidade, superioridade do LM sobre a fórmula artificial, custo do AM, efeito protetor do AM, proteção contra infecções/alergias, efeito protetor do AM no câncer de mama e colo de útero.	Biológica Sociocultural	4.b
Mohamad^(28)^ 2019, Malásia	**Desenho:** transversal. **Amostra:** 162 estudantes de medicina e odontologia do último ano da *Universiti Sains Malaysia* (75%). **ICD:** questionário estruturado e autoadministrado (11 itens desenvolvido e validado por Che Muzaini *et al*.^(13)^), utilizado com permissão dos autores, com quatro partes: 1) perguntas sobre características demográficas dos participantes; 2) intenções futuras dos participantes de amamentar e exposição à amamentação exclusiva; 3) conhecimento sobre AM; 4) atitudes dos estudantes em relação à amamentação exclusiva.	Avaliar o conhecimento, as atitudes, a exposição e as intenções futuras em relação ao AM exclusivo entre estudantes de medicina e odontologia do último ano da *Universiti Sains Malaysia*, Kelantan, Malásia.	Recomendações AM, vantagens do AM (mãe/criança), problemas do AM (traumas mamilares, ingurgitamento, icterícia), duração e início e frequência da mamada, expressão do LM (ordenha, armazenamento e oferta).	Biológica Manejo Política Sociocultural	4.b
Altwalbeh^(29)^ 2021, Jordânia	**Desenho:** estudo descritivo de corte transversal, amostragem de conveniência. **Amostra:** 72 estudantes de obstetrícia **ICD:** versão curta do Questionário Australiano de Conhecimento e Atitude em Amamentação Materna (ABKAQ-SF), desenvolvido por Brodribb. Questionário adaptado (48 itens), com características demográficas dos estudantes, sua confiança percebida em suas habilidades e sua satisfação com relação ao conteúdo do AM no currículo acadêmico^(8)^, além de atitudes de AM^(18)^, escala Likert, conhecimento dos participantes sobre o AM^(22)^ com o tipo de resposta “correto/incorreto” e “não sei”, e 11 delas são itens falsos.	Avaliar o conhecimento e as atitudes de estudantes de obstetrícia em relação ao AM.	Benefícios e a fisiologia do AM e manejo do AM.	Biológica Manejo	4.b
Biggs^(30)^ 2020, Reino Unido	**Desenho:** transversal, com coleta *online*. **Amostra:** 411 estudantes do 5º e 6º anos de medicina de 22 universidades do Reino Unido. **ICD:** dois questionários *online*, um para avaliar a educação sobre amamentação fornecida no currículo (benefícios do AM, diferença entre leite materno e fórmula láctea) e outro para determinar o conhecimento e as percepções dos alunos sobre a sua confiança no apoio às mães que amamentam, o papel percebido dos médicos no apoio à amamentação, aspirações profissionais e interesse em receber ensino adicional sobre amamentação.	Suprir essa lacuna de conhecimento e explorar a percepção dos estudantes sobre sua prontidão para apoiar o AM.	Propriedades imunológicas, proteção contra doenças infecciosas, câncer de ovário, mama na mãe, diabetes tipo I e II na vida adulta, razões médicas para indicar o uso da fórmula e o impacto ambiental.	Biológica Sociocultural	4.b
Cervera-Gasch^(31)^ 2021, Espanha	**Desenho:** estudo observacional, descritivo, transversal e multicêntrico. **Amostra:** 684 estudantes de enfermagem de três universidades. **ICD:** questionário AprendLac, validado para o espanhol, com 21 questões de múltipla escolha: 1) sociodemográfica e acadêmica; 2) conhecimentos.	Estudar o nível de conhecimento de enfermagem de estudantes de enfermagem em AM em três universidades públicas espanholas; explorar quais variáveis estão relacionadas à aquisição desse conhecimento.	Recomendações do AM de acordo com a OMS, alimentação artificial, cuidados com mastites, alimentação virtual, colostro, AM na primeira hora, frequência de alimentação, higiene dos seios, perda de peso do recém-nascido, mamanalgesia, avaliação da sucção ineficiente, pega e posicionamento do bebê ao seio, lactogênese, cuidados canguru, benefícios do AM para o bebê e para a mãe, e AM de prematuros.	Biológica Manejo Política ACS	4.b
Yakovlev^(32)^ 2021, Rússia	**Desenho:** estudo multicêntrico realizado em oito universidades na Rússia. **Amostra:** 1.088 estudantes de medicina do 5º e 6º anos, estagiários e residentes de pediatria. **ICD:** questionário desenvolvido por Yakovlev *et al*. com 43 questões, sendo 32 perguntas fechadas, nove abertas e dois parcialmente.	Analisar o conhecimento (e sua dinâmica ao longo da última década) de estudantes de medicina, estagiários e residentes sobre o apoio e promoção do AM na Federação da Rússia.	Preparação para a AM, apoio à nutriz, conhecimentos errôneos, fisiologia da lactação, alimentação infantil, recomendações da OMS, estimulação da lactação, publicidade de fórmula.	Biológica Manejo Política Sociocultural ACS	4.b

*Nota: ID - identificação; ICD - instrumento com dimensões; LM - lactação materna; AM - aleitamento materno; OMS - Organização Mundial da Saúde; ACS - alimentação complementar saudável.*

*Nível de evidência: 2e - Estudos prospectivamente controlados de quase-experimentos; 3d - Estudo de caso-controle; 4b - Estudo transversal descritivo ou observacional (Peters, 2015).*

No presente estudo, será considerado o conhecimento da temática do AM, a partir de seis dimensões, que englobam a multiplicidade de aspectos envolvidos para o sucesso da prática da amamentação, como sociocultural, biológica, manejo clínico da lactação, política de AM (nacional e internacional), legislação e proteção do AM, e ACS.

A classificação dos achados dos estudos primários nas seis dimensões temáticas foi realizada pelos autores com base na análise criteriosa dos objetivos dos estudos, dos conteúdos dos instrumentos de coleta de dados e dos principais resultados reportados. Cada estudo foi categorizado na(s) dimensão(ões) cujos temas eram o foco central da investigação e dos resultados apresentados, buscando a maior aderência possível entre o conteúdo do artigo e as definições das dimensões propostas para esta revisão.

A opção pela classificação das dimensões supracitadas tem como justificativa ser um ponto de partida para se estruturar uma metodologia de ensino que seja o mais abrangente possível, considerando a amamentação enquanto um fenômeno existencial que se constitui pelo sinergismo entre a natureza e a cultura^(14)^.

Após a categorização dos estudos, os dados foram sintetizados para a análise descritiva de acordo com o ano de publicação, idioma, local onde o estudo foi realizado, objetivo, tipo de estudo e nível de evidência. Por fim, foram realizadas a análise e síntese dos artigos obtidos de forma descritiva para subsidiar a apresentação dos resultados, discussão e conclusões deste estudo de revisão.

## RESULTADOS

A busca nas bases de dados resultou em um total de 967 artigos; desses, 96 artigos foram excluídos, pois eram duplicados. Após essa etapa, 871 artigos foram avaliados através dos títulos e resumos, com base nos critérios estabelecidos, identificando 25 artigos para leitura na íntegra, ainda considerando os critérios de inclusão e exclusão, sendo selecionados 17 artigos.

Na sequência, apresentaram-se o processo de seleção dos artigos e a descrição das razões de exclusão, utilizando o fluxograma criado pelos autores para a presente revisão (Figura 1):

**Figura 1:** Fluxograma das etapas de identificação, seleção e inclusão dos estudos, adaptado do Preferred Reporting Items for Systematic Reviews and Meta-Analyses

*Fonte: Page MJ et al.^(15)^*

O Quadro 2 apresenta o perfil da amostra de artigos selecionados para análise.

No Quadro 2, identificamos que, dos artigos incluídos, os países predominantes foram os Estados Unidos, com quatro artigos (23%), seguido do Brasil, com três artigos (17%). Em termos de representação continental, a América e a Ásia contribuíram com sete artigos cada (41%), a Europa, com dois (12%), e a África, com um (6%). O idioma predominante foi o inglês.

Quanto ao tipo de instrumento de coleta de dados, oito utilizaram questionários próprios e nove informaram ter adaptado do instrumento original com autorização, sendo AprendLac^(31)^, Brodribb^(21,24,29)^, Che Muzaine^(28)^, Kavanagh^(27)^, Smith^(18)^, e Wlliams e Hammer^(17)^.

Em relação à abordagem das dimensões, seis artigos abordaram duas dimensões^(19,22,25,27,29,30)^; seis abordaram três dimensões^(16-18,20,24,26)^; quatro abordaram quatro dimensões^(21,23,28,31)^; e apenas um abordou cinco dimensões^(32)^. Nenhum questionário teve uma abordagem unidimensional, porém a dimensão com maior ocorrência de abordagem foi a biológica, seguida do manejo da lactação.

No que se refere à dimensão biológica, os estudos incorporam a importância do AM para prevenir doenças na criança^(21,23,24,27)^, aspectos relacionados a: anatomia e fisiologia da lactação^(17,20-24,29,31)^; benefícios do leite materno^(17,18,20-24,28,29,31)^; condições maternas que podem afetar a amamentação^(7)^; uso de fármacos no período de lactação^(20,21,23,24)^; efeito protetor do AM na prevenção do câncer de mama e colo de útero^(21,24,27)^; propriedades do leite materno^(18,20,31)^; superioridade sobre a fórmula artificial^(21,24,27)^; alterações metabólicas e risco de obesidade^(27)^.

Com o foco em tais temáticas, notou-se uma preocupação dos pesquisadores em identificar o conhecimento dos estudantes sobre os aspectos relacionados ao funcionamento da mama, as estruturas implicadas na lactação, a composição do leite humano e a lactação em condições especiais, a fim de que este futuro profissional possa se sentir mais seguro e preparado para o manejo da lactação.

Nesse sentido, os estudos que abordaram a dimensão do manejo da lactação focaram em temas como: AM de prematuros^(31)^; AM e vínculo^(24)^; avaliação da sucção ineficiente do lactente^(17,31)^; barreiras, dificuldades e contraindicações à amamentação^(16,17)^; desmame^(20,23)^; duração da mamada^(19,28)^; expressão de leite materno (ordenha, armazenamento e oferta)^(17,28)^; frequência da mamada^(16,21,28)^; higiene dos seios^(31)^; início do AM^(17,19,28)^; mamanalgesia^(31)^; técnica da amamentação^(17,19,20,22,25,29,31)^; manejo da mastite^(31)^; parâmetros de amamentação eficaz^(18)^; pega e posicionamento do bebê ao seio^(25,31)^; perda de peso do recém-nascido^(31)^; prevenção e manejo de traumas mamilares^(17,22,28)^; uso de intermediários/auxiliares de amamentação^(2)^; e suplementação do AM^(16,19,21,24,31)^.

A abordagem da dimensão política se restringiu à política internacional, marcadamente pela recomendação da Organização Mundial da Saúde (OMS) sobre a duração da amamentação, que é “aleitamento materno exclusivo por seis meses e continuado por dois anos ou mais”^(20,23,28,31,32)^ e ao Método Canguru^(31)^, que constitui uma política voltada à qualificação do cuidado do neonato e sua família^(33)^.

A dimensão legislação não foi contemplada em nenhum dos estudos analisados. Embora, em alguns deles, a palavra “proteção ao AM” estivesse presente, associava-se ao manejo ou à dimensão sociocultural. Nos dez artigos que inseriram a dimensão sociocultural em seus instrumentos, o destaque foi para a autoimagem “mulheres com seio pequeno não produzem leite suficiente”^(26)^, a amamentação enquanto um valor humano^(23,26,28)^, conselhos sobre alimentação artificial^(31)^, amamentação em público compreendida à luz da cultura de um povo^(16,18,21,24)^ e custo do AM^(27)^.

Em relação à dimensão da ACS, verificou-se que os estudos estavam centrados no grau de conhecimento acerca da introdução alimentar^(16,21,24)^ e do manejo da alimentação infantil, após o período do desmame^(26)^.

## DISCUSSÃO

Esta revisão mostrou que a maioria dos estudos que preencheram os critérios de inclusão foram produzidos na Ásia e na América (Estados Unidos e Brasil), e obtiveram evidências a partir do delineamento de pesquisas quantitativas do tipo transversal.

Embora seja uma preocupação governamental estabelecer diretrizes curriculares para garantir o conhecimento, competências e habilidades específicas de estudantes ao longo do seu processo de formação acadêmica^(34)^, nota-se que ainda é incipiente a produção científica internacional e brasileira acerca do conhecimento de graduandos da área da saúde sobre a temática do AM em suas múltiplas dimensões.

Estudos que têm como objetivo o conhecimento de estudantes de graduação sobre AM deveriam partir da compreensão de que, para uma adequada formação profissional, e, por conseguinte, uma assistência segura e de qualidade, há um mínimo relevante de conteúdos teóricos e práticos que possibilitarão ao estudante a aquisição de habilidades e competências que, sendo oferecidas durante a graduação, irão impactar o sucesso da amamentação.

Esses achados reforçam a necessidade premente de que as instituições de ensino superior e os colegiados de curso na área da saúde revisitem e atualizem seus currículos para aprofundar a abordagem das múltiplas dimensões que envolvem o AM. É fundamental que a formação dos futuros profissionais vá além dos aspectos biológicos e de manejo clínico, incorporando conhecimentos sólidos sobre os determinantes sociais, culturais, econômicos, legislativos e políticos que impactam a decisão e a prática da amamentação. A integração desses saberes, alinhada às Diretrizes Curriculares Nacionais para os cursos de saúde e aos princípios do Sistema Único de Saúde (SUS), capacitará o egresso a atuar de forma mais eficaz na promoção, proteção e apoio ao AM, considerando a complexidade do fenômeno e o contexto de vida das famílias. Isso implica a adoção de metodologias de ensino ativas e problematizadoras que estimulem a reflexão crítica e o desenvolvimento de competências para a prática interprofissional e colaborativa.

Nessa perspectiva, observa-se que tais conhecimentos, habilidades e atitudes devem estar pautados no paradigma multifatorial relacionado ao AM, no qual diferentes saberes devem ser articulados entre si. Associado a isso, é importante considerar que os modos como aprendemos os conteúdos na universidade não se dão de maneira linear, nem uniforme.

Tal afirmativa vem ao encontro dos estudos que mostram que o modo de ensinar e de aprender está intrinsicamente relacionado às competências desenvolvidas ao longo da formação, sejam estas competências cognitivas, onde o saber propriamente dito é adquirido em escolas, livros, universidades, trabalho, veículos de comunicação ou de pessoa a pessoa; competências práticas, pautadas no saber fazer, executar uma ação a partir da tomada de decisão; e competências morais, que levam o indivíduo a pôr em prática um determinado conhecimento, a partir de seus desejos, crenças, valores e vivências dirigidas a um determinado objeto, situação ou pessoa^(34,35)^.

Como reflexo do conhecimento acima descrito, as questões referentes ao manejo da lactação constituem uma importante dimensão, pois abrangem as principais condutas e orientações que devem ser fornecidas às mães que amamentam. Estas podem se deparar com problemas ou dificuldades desde o início do período da lactação e para a prevenção do desmame precoce. Além das orientações individuais, deve-se contar com legislação e políticas em prol da amamentação.

Observou-se que ainda é incipiente a produção de estudos acerca do conhecimento de estudantes de graduação na área da saúde sobre AM com foco nas dimensões política e legislação. A dimensão legislação não foi contemplada em nenhum dos estudos analisados, enquanto a dimensão política se restringiu majoritariamente às recomendações da OMS e ao método canguru^(36)^. Nesse sentido, ressalta-se a fundamental importância do conhecimento por parte dos futuros profissionais de saúde acerca das estratégias e ações inseridas nas políticas nacionais, internacionais de AM e das bases legais que visam à proteção, promoção e apoio ao AM. O aprendizado sobre as recomendações já estabelecidas em decretos, leis e resoluções, como a Norma Brasileira de Comercialização de Alimentos para Lactentes e Crianças de Primeira Infância, a licença maternidade/paternidade e a Rede Brasileira de Bancos de Leite Humano, é de suma importância para o suporte e a prática segura da amamentação, sendo arcabouço para a elaboração e implementação de políticas e programas eficazes. A compreensão do papel do profissional de saúde na defesa e no cumprimento desta legislação é essencial para a criação de um ambiente favorável à amamentação.

A amamentação é tal qual um prisma que, ao lhe projetar luz, novas formas se evidenciam, podendo ser compreendida do ponto de vista da economia, religião, política, mercado, relações sociais, gênero, ciência, ética e bioética, todas entrelaçando-se em uma trama, convergindo para seu sucesso ou o desmame de forma equidistante. O que vai pender a balança para um ou outro lado é o investimento que fazemos, quer seja como indivíduo, que vivencia o fenômeno, que seja como família, profissionais de saúde, sociedade e Estado.

Por essa razão, é fundamental compreender que a decisão sobre amamentar não depende unicamente do conhecimento de seus benefícios, ou de processos fisiológicos como a funcionalidade das glândulas maternas e do sistema estomatognático da criança^(37)^.

Nessa perspectiva, vale considerar a dimensão sociocultural da amamentação, que se refere a fatores ambientais, tradições familiares ou práticas sociais e cotidianas, crenças populares, e a influência de mensagens transmitidas nos meios de comunicação, na literatura, novelas, filmes e postagens nas redes sociais, que podem influenciar e impactar a decisão materna de amamentar^(38)^. Os estudos incluídos que abordaram esta dimensão destacaram aspectos como autoimagem corporal (“mulheres com seio pequeno não produzem leite suficiente”), amamentação como valor humano, conselhos sobre alimentação artificial e amamentação em público sob a ótica cultural. Estes e outros aspectos reforçam o quanto o conhecimento acerca das questões socioculturais é necessário na formação do profissional de saúde, contribuindo para uma cosmovisão que permita a compreensão da amamentação de forma ampliada, para além da dupla mãe-bebê, e com isso, posicionar-se de forma mais efetiva como membro da rede de apoio da mulher que amamenta. A sensibilidade para identificar e abordar as barreiras socioculturais é crucial para oferecer um cuidado individualizado e respeitoso.

Apesar do enfoque dado na promoção do AM exclusivo nos primeiros seis meses de vida da criança, cabe ao estudante conhecer também as questões referentes à dimensão da ACS^(16,21,39,40)^. Verificou-se que os estudos analisados estavam centrados no grau de conhecimento acerca do momento correto, da forma de introdução alimentar e do manejo da alimentação infantil após o período do desmame. Entretanto, outros aspectos cruciais da alimentação complementar, como a qualidade nutricional dos alimentos, as práticas alimentares familiares, a segurança alimentar e nutricional e a influência de fatores socioculturais na aceitação de determinados alimentos, foram pouco explorados na produção científica incluída nesta revisão.

Corroborando tais achados, investigações realizadas com profissionais de saúde apontam para a relevância das orientações nutricionais e de higiene, que devem ser fornecidas à nutriz para garantir uma nutrição adequada após o término da amamentação exclusiva do bebê e, consequentemente, um crescimento e desenvolvimento satisfatórios durante os primeiros anos de vida^(39)^. A formação dos futuros profissionais de saúde deve, portanto, contemplar de forma mais abrangente os diferentes aspectos da ACS, capacitando-os a oferecer orientações completas e culturalmente sensíveis às famílias.

### Limitações de estudo

Como limitação do presente estudo, considera-se o número reduzido de artigos obtidos na busca nas bases de dados pesquisadas. Além disso, a ausência de um protocolo detalhado para a avaliação da qualidade metodológica dos estudos incluídos e para a categorização dos achados nas dimensões temáticas pode ser considerada uma limitação, embora a classificação tenha sido realizada criteriosamente pelos autores com base no conteúdo dos artigos. No entanto, acredita-se que tais fatos não comprometeram a qualidade da investigação, e os resultados encontrados apontaram para a necessidade de estudos futuros com a finalidade de ampliar a discussão acerca dos conhecimentos, atitudes e práticas de graduandos sobre a temática do AM.

### Contribuições para as áreas da enfermagem e saúde

Verificou-se a importância de se investir na formação acadêmica dos estudantes de graduação em enfermagem e de outras áreas da saúde, para que, atuando de forma interprofissional, possam exercer uma escuta ativa e representarem um suporte para as famílias que vivenciam o processo de amamentação. Desta forma, destaca-se o papel dos coordenadores de curso, bem como dos docentes, de priorizar conteúdos pertinentes à multidimensionalidade da prática do AM. Ressalta-se ainda a importância dos futuros profissionais da rede de saúde, que serão aqueles responsáveis pelo apoio necessário às mães que desejarem amamentar seus filhos.

## CONCLUSÕES

A presente revisão integrativa da literatura possibilitou a análise da produção científica acerca do conhecimento de graduandos da área da saúde sobre AM, a partir das dimensões intrínsecas a esta temática. Os estudos responderam à pergunta norteadora e apontaram para o fato que o conhecimento dos estudantes se restringiu predominantemente às dimensões biológica e de manejo da lactação. Em contrapartida, as dimensões socioculturais, ACS, legislação e política de proteção ao AM foram pouco exploradas nos estudos analisados, evidenciando lacunas significativas no conhecimento dos futuros profissionais nessas áreas cruciais.

Considera-se que a formação do estudante na temática AM deva contemplar, de forma equitativa e aprofundada, as seis dimensões apresentadas: biológica; manejo clínico; legislação; política de proteção ao AM; sociocultural; e ACS. A integração desses saberes é fundamental, a fim de propiciar um cuidado centrado não apenas nas orientações e técnicas, mas que envolva a compreensão do contexto social, cultural, político e legal, bem como a multiplicidade de fatores implicados no processo da amamentação.

Diante das lacunas de conhecimento identificadas, particularmente nas dimensões menos abordadas pelos estudos, faz-se imperativo que as instituições de ensino superior promovam revisões curriculares que assegurem a inclusão transversal e aprofundada da temática da amamentação em suas múltiplas facetas. Sugere-se a incorporação de metodologias de ensino ativas e participativas que incentivem a reflexão crítica, o desenvolvimento de habilidades práticas e a sensibilidade para as questões socioculturais e emocionais que envolvem a amamentação. É igualmente essencial integrar o ensino sobre AM às políticas de saúde vigentes, preparando os estudantes para atuar em consonância com as diretrizes nacionais e internacionais, para se tornarem agentes de transformação na promoção e proteção do AM no SUS.

O número limitado de publicações internacionais e nacionais que avaliam o conhecimento dos estudantes sobre a amamentação em todas as suas dimensões evidencia a importância do desenvolvimento de futuros estudos com desenhos metodológicos diversos e robustos, incluindo pesquisas de intervenção e qualitativas, para ampliar a compreensão das formas de ensino mais eficazes sobre a temática nos cursos de graduação da área da saúde e investigar o impacto de diferentes abordagens pedagógicas no conhecimento e nas competências dos futuros profissionais.

## Data Availability

Os dados de pesquisa só estão disponíveis mediante solicitação.
